# Supplementary material for: Integrated Analysis of Cytokine Profiles in Malaria Patients Discloses Selective Upregulation of TGF-β1, β3, and IL-9 in Mild Clinical Presentation
Source: Int J Mol Sci. 2022 Oct 21;23(20):12665. doi: 10.3390/ijms232012665 (PMC9603849; doi:10.3390/ijms232012665)
Supplement: Supplementary file 1 [file ijms-23-12665-s001.zip › Supplementary Tables.pdf]

Supplementary Table S1: Demographic and clinical details of study participants

Characteristics	Control	Uncomplicated Malaria	Severe anaemic Malaria	T-test	P- Value
No. of participants	58	186	80		
Sex (M:F)	29:29	100:86	50:30		
Age (years)	30(10-50)	35(5-65)	36(5-68)		
Mean of parasites density/ µl blood	0	3100	9100	12.356	p<0.0001
Upper 95% CI of mean of parasites density/µl blood	0	41002	122185		
Mean of Hemoglobin conc. (g/dl)	14.02	13.5	5.44		

Supplementary Table S2: Cytokines Mean ± SEM

Cytokines & Immunoglobulins	Malaria clinical manifestations	Mean ± SEM
IL-4	Control	95 ± 105
	Mild infection	13234 ± 63.95
	Severe infection	1045 ± 60.62
IL-6	Control	310 ± 162
	Mild infection	772 ± 41.79
	Severe infection	4139 ± 291.8
IL-7	Control	52 ± 20
	Mild infection	1705 ± 45.81
	Severe infection	643 ± 30.58
IL-9	Control	1676 ± 560
	Mild infection	6365 ± 134.7

	Severe infection	1876 ± 57.14
IL-10	Control	462 ± 218
	Mild infection	3037 ± 130.2
	Severe infection	1239 ± 67.83
IL-13	Control	720 ± 360
	Mild infection	1892 ± 40.62
	Severe infection	6149 ± 143.8
IL-17	Control	67 ± 38
	Mild infection	2070 ± 62.76
	Severe infection	839 ± 50.79
IL-27	Control	922 ± 1007
	Mild infection	3869 ± 2698
	Severe infection	1776 ± 1698
TGF-ß1	Control	174 ± 197
	Mild infection	1577 ± 64.23
	Severe infection	<20
TGF-ß3	Control	<20
	Mild infection	4209 ± 173.5
	Severe infection	391± 41.21
IFNγ	Control	209 ± 90
	Mild infection	476 ± 23.17
	Severe infection	2713 ± 89.38
TNF	Control	208 ± 128
	Mild infection	377 ± 17
	Severe infection	3433 ± 107
