# Supplementary figures and images for: Integrated Analysis of Cytokine Profiles in Malaria Patients Discloses Selective Upregulation of TGF-β1, β3, and IL-9 in Mild Clinical Presentation
Source: Int J Mol Sci. 2022 Oct 21;23(20):12665. doi: 10.3390/ijms232012665 (PMC9603849; doi:10.3390/ijms232012665)

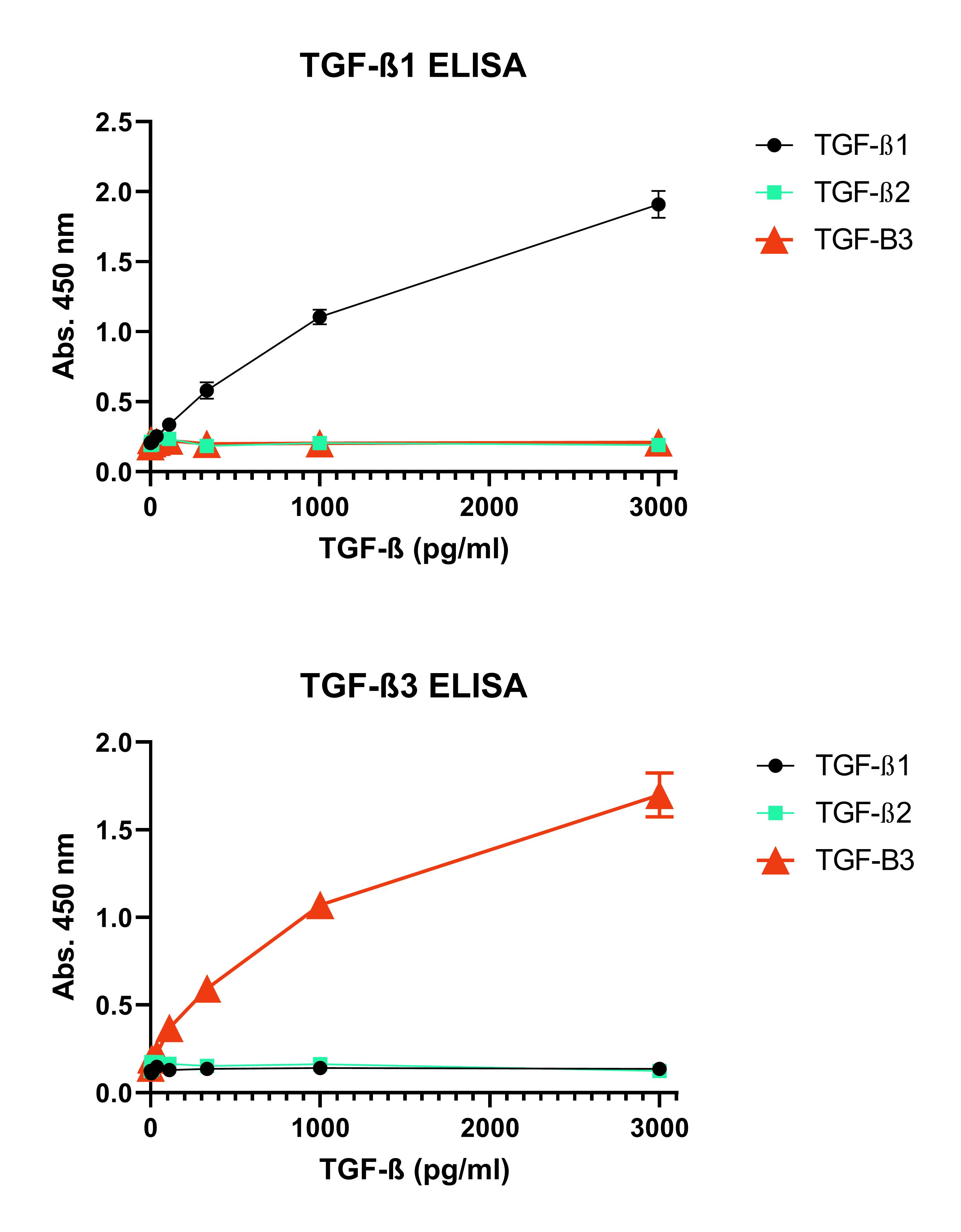

Supplement: Supplementary file 1 [file ijms-23-12665-s001.zip › Supp Figure S1.jpg]

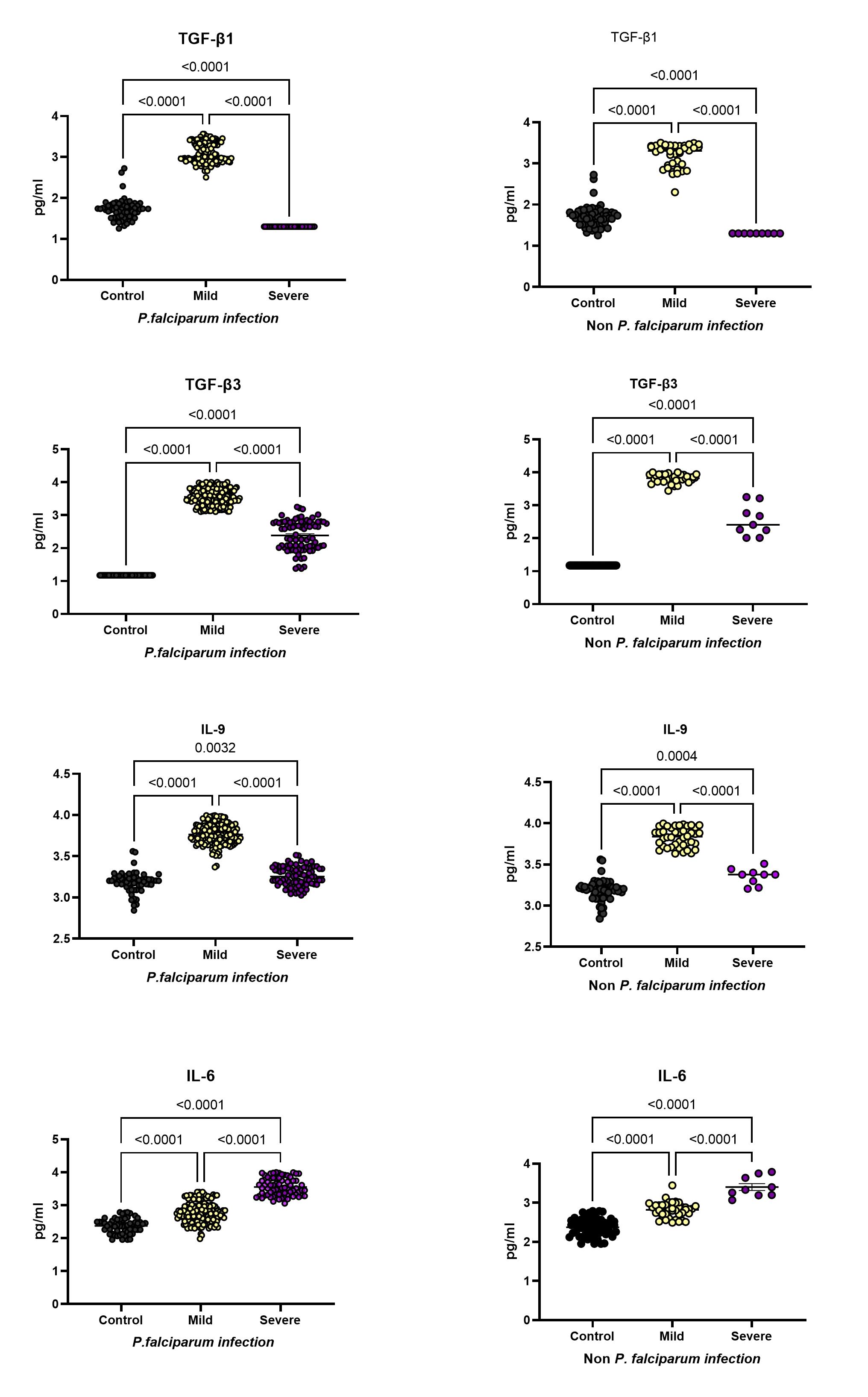

Supplement: Supplementary file 1 [file ijms-23-12665-s001.zip › Supp Figure S2.jpg]

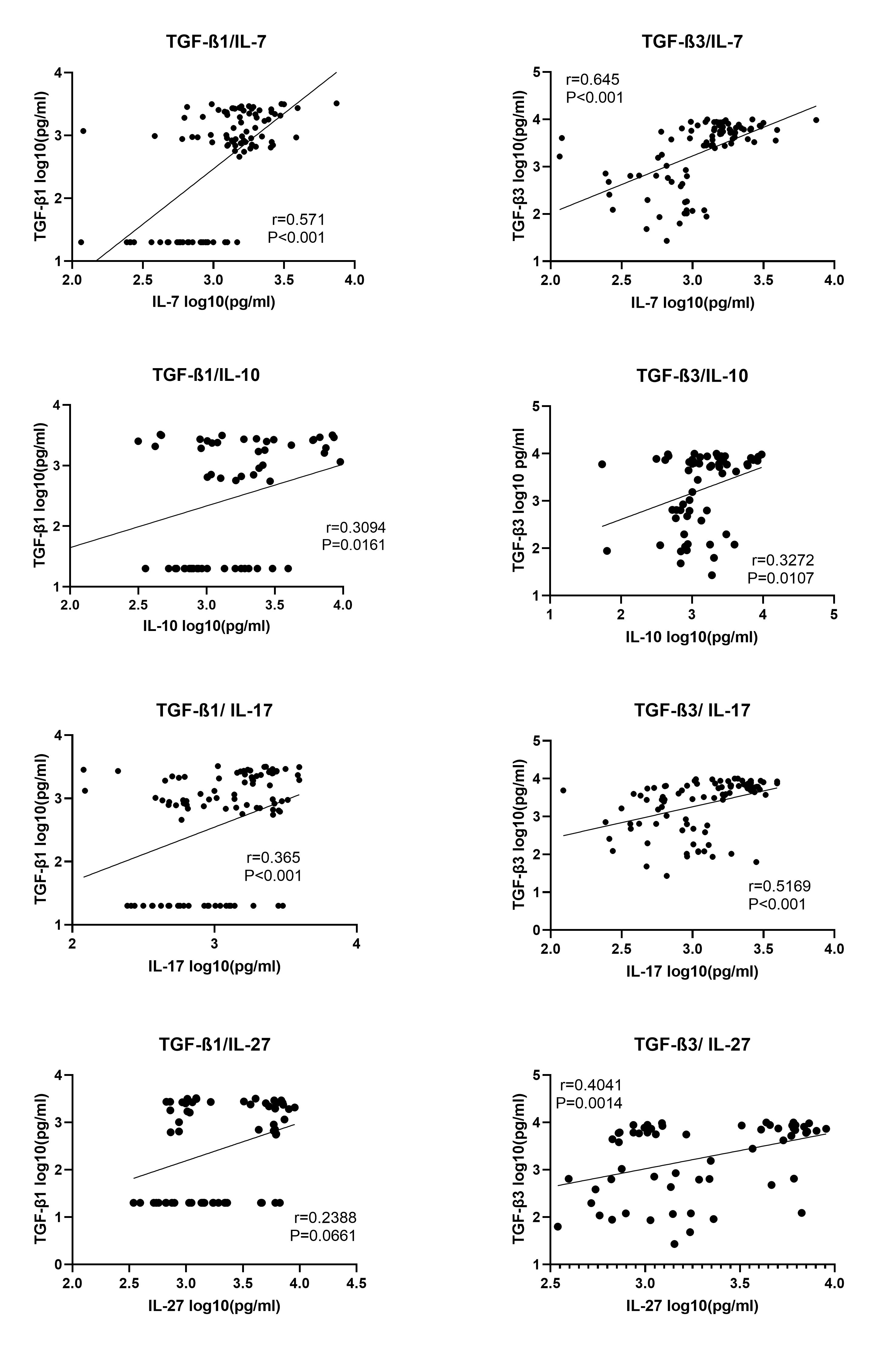

Supplement: Supplementary file 1 [file ijms-23-12665-s001.zip › Supp Figure S3.jpg]

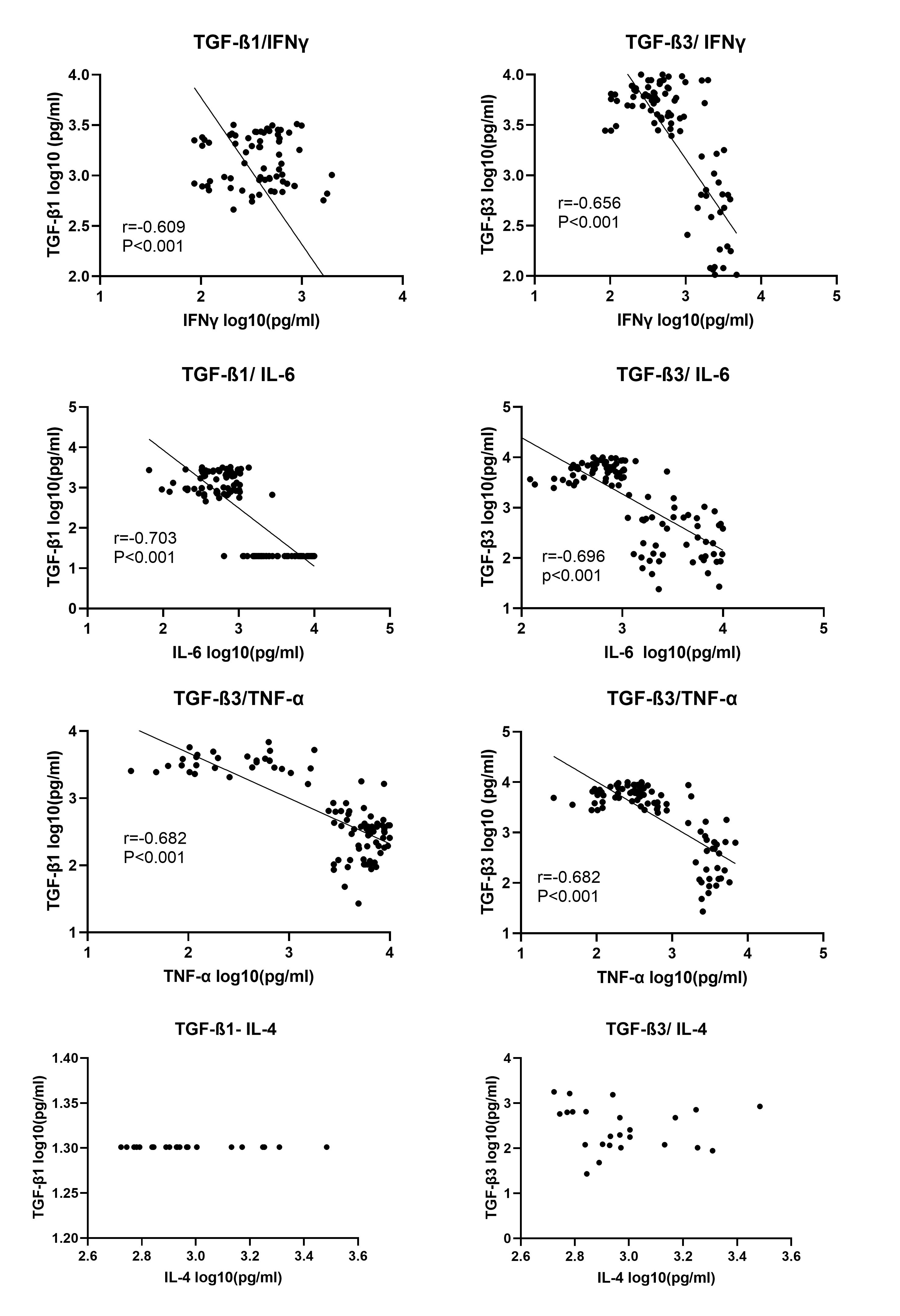

Supplement: Supplementary file 1 [file ijms-23-12665-s001.zip › Supp Figure S4.jpg]
